# Considering Comorbidities and Individual Differences in Testing a Gaming Behavioral Activation App for Perinatal Depression and Anxiety: Open Trial Pilot Intervention Study

**DOI:** 10.2196/59154

**Published:** 2025-01-14

**Authors:** Gabriella E Hamlett, Chloe Schrader, Craig Ferguson, Lauren A Kobylski, Rosalind Picard, Joseph J Locascio, Richard J McNally, Lee S Cohen, Rachel Vanderkruik

**Affiliations:** 1 Center for Women’s Mental Health, Massachusetts General Hospital, Boston, MA, United States; 2 Department of Psychology, Harvard University, Cambridge, MA, United States; 3 MIT Media Lab, Massachusetts Institute of Technology, Cambridge, MA, United States; 4 Department of Psychological & Brain Sciences, George Washington University, Washington, DC, United States; 5 Harvard Catalyst Biostatistical Consulting Group and Department of Neurology, Massachusetts General Hospital, Boston, MA, United States; 6 Department of Neurology, Harvard Medical School, Boston, MA, United States; 7 Department of Psychiatry, Harvard Medical School, Boston, MA, United States

**Keywords:** perinatal anxiety, perinatal depression, behavioral activation, digital mental health, mobile phone

## Abstract

**Background:**

There is increasing interest in the development of scalable digital mental health interventions for perinatal populations to increase accessibility. Mobile behavioral activation (BA) is efficacious for the treatment of perinatal depression; however, the effect of comorbid anxiety and depression (CAD) on symptom trajectories remains underexplored. This is important given that at least 10% of women in the perinatal period experience CAD.

**Objective:**

We assessed whether there were differences in symptom trajectories in pregnant participants with CAD as compared to those with depression only (ie, major depressive disorder [MDD]) during intervention with a BA mobile gaming app.

**Methods:**

Pregnant adults with either CAD (n=10) or MDD (n=7) used a BA app for 10 weeks and completed biweekly symptom severity questionnaires for depression and anxiety. We assessed whether baseline diagnoses were associated with differential symptom trajectories across the study with mixed effects longitudinal models.

**Results:**

When controlling for baseline symptoms, results revealed a significant interaction between baseline diagnosis and the quadratic component of study week on anxiety (β=.18, SE 0.07; *t*_62_=2.61; *P*=.01), revealing a tendency for anxiety in the CAD group to increase initially and then decrease at an accelerated rate, whereas MDD symptoms were relatively stable across time. There was a significant effect of linear time on depression (β=−.39, SE 0.11; *t*_68_=−3.51; *P*=.001), showing that depression declined steadily across time for both groups. There was a significant effect of baseline diagnosis on depression (β=−8.53, SE 3.93; *t*_13_=−2.17; *P*=.05), suggesting that those with MDD had higher follow-up depression compared to those with CAD when holding other predictors constant.

**Conclusions:**

The app was beneficial in reducing depression symptoms in perinatal individuals with different comorbidity profiles. With respect to anxiety symptom trajectories, however, there was more variability. The app may be especially effective for the treatment of anxiety symptoms among individuals with CAD, as it encourages in-the-moment ecologically relevant exposure to anxiety-provoking stimuli. Despite no significant group difference in baseline anxiety symptoms, the MDD group did not have a significant reduction in their anxiety symptoms across the study period, and some individuals had an increase in anxiety. Findings may point to opportunities for the augmentation of BA gaming apps for those with MDD to more effectively target anxiety symptoms. Overall, findings suggest there may be value in considering comorbidities and individual variations in participants when developing scalable mobile interventions for perinatal populations.

## Introduction

During the perinatal period, 19%‐40% of women experience anxiety or depression, and at least 10% experience comorbid anxiety and depression (CAD [1,2]). Despite its prevalence, CAD in perinatal populations is relatively underexplored [3]. CAD is associated with greater symptom severity, decreased treatment response, and longer episode duration [2-5]. Therefore, more research is needed to assess treatment considerations for perinatal individuals with CAD.

Behavioral activation (BA) is a behavioral intervention that aims to boost engagement in pleasurable activities, emphasizing value-driven behaviors and avoidance reduction, and is efficacious for the treatment of perinatal depression and anxiety [6]. While there is a strong need for the treatment of anxiety and depression in perinatal populations, access to treatment remains challenging and thus further highlights the need for scalable treatments that are accessible for use in ecologically valid settings [7]. Due to accessibility difficulties and maternity health care deserts [8], there is increasing interest in the development of scalable digital mental health interventions for perinatal populations [9-13].

Vanderkruik et al’s [14] pilot study on the feasibility and acceptability of a BA gaming app for pregnant women suggests its potential to decrease depression symptoms during pregnancy. However, the effect of comorbidity on symptom trajectories remains unexplored, and few studies focus on individual differences in mobile BA treatment outcomes [14,15]. In this pilot study, we take a hypothesis-generating approach to assess differences in anxiety and depression symptom trajectories in participants with either CAD or major depressive disorder (MDD).

## Methods

### Recruitment

Participants (pregnant adults, native English speakers, smartphone users, and at least moderately depressed [14]) were recruited throughout the United States between 2021 and 2022 via clinician referrals, the Massachusetts General Hospital Center for Women’s Mental Health website [16], and social media advertising. Ineligibility criteria included imminent risk of self-harm, current substance abuse, psychotic disorder, or active mania. All participants (n=18) met the *Diagnostic and Statistical Manual of Mental Disorders, Fifth Edition* (*DSM-5*) criteria for a current major depressive episode, 10 of whom additionally met criteria for generalized anxiety disorder (GAD) as assessed by the Mini International Neuropsychiatric Interview (MINI [17]). One participant dropped from the study due to active substance use, resulting in a final sample of 17.

Participants were instructed to use the app for the 10-week study period and invited to complete daily “Adventures” (eg, exercising and cleaning) in their real life to earn points and progress in the game. Participants completed surveys for depression and anxiety symptoms biweekly over the 10-week study period [14].

### Ethical Considerations

This study is a secondary analysis of deidentified data from the parent study. All original study procedures were approved by the Mass General Brigham institutional review board (2021P001400). All participants provided informed consent in the parent study, which stipulated that deidentified data may be analyzed in secondary analyses without additional consent. Participants were not provided compensation for their participation.

### Measures

Eligibility was assessed at baseline with the MINI [17], a semistructured interview conducted by a trained research assistant [14]. Participant grouping (CAD vs MDD) was determined at baseline using MINI diagnostic criteria. The CAD group included individuals who met *DSM-5* criteria for GAD (excessive, difficult to control anxiety and worry for most days for at least 6 months and 3 of the following symptoms: restlessness, being easily fatigued, difficulty concentrating, irritability, muscle tension, and sleep disturbance [18]) and MDD (a depressed mood for most of the day or anhedonia for at least 2-weeks, as well as 5 of the following symptoms: significant unintentional weight or appetite change, insomnia or hypersomnia, psychomotor agitation or retardation, fatigue, feelings of worthlessness or inappropriate or excessive guilt, decreased ability to concentrate or make decisions, and recurrent thoughts of death or suicidal ideation [19]). Those in the MDD group only met the criteria for MDD. Self-reported anxiety and depression symptoms within the past two weeks were measured with the Generalized Anxiety Disorder-7 (GAD-7 [18]) and Patient Health Questionnaire-9 (PHQ-9 [14]), respectively. Study completers were defined as participants who completed the final assessment at the end of the 10-week study period. As published previously [14], app engagement was measured by assessing days using the app (ie, the number of days a participant logged on) and activities completed (ie, the total number of activities a participant completed in the game).

### Statistical Analysis

Given the scope of this pilot study, we chose a hypothesis-generating approach with the intent of informing future research, though we expected to see less favorable depression and anxiety symptom trajectories (according to PHQ-9 and GAD-7, respectively) for CAD relative to the MDD group. The analyses were conducted in R (version 4.3.0; R Core Team). Demographic variables, group differences in reductions of anxiety and depression symptoms, app engagement, study completion, and individual symptom trajectories by group were analyzed with descriptive statistics. Independent group *t* tests (2-tailed) explored differences in baseline measures of depression and anxiety by baseline diagnosis.

To assess individual variation in depression and anxiety symptom decreases over time within each group, we plotted individual participant data to visualize trajectories of change for anxiety and depression symptoms across time. To determine whether treatment outcomes changed differentially across time by baseline diagnosis while controlling for baseline depression and anxiety symptom levels, we used mixed effects longitudinal models. In separate models, PHQ-9 and GAD-7 (hereafter referred to as depression and anxiety symptoms, respectively) were the dependent variables. We controlled for baseline depression and anxiety to ensure that trajectories were due to the app and diagnostic group rather than differences in baseline symptom levels. Fixed predictors were baseline diagnosis group (CAD vs MDD), time (linear and quadratic components of the week in study), baseline depression or anxiety symptoms for the analysis of their respective dependent variables, and all 2 and 3-way interactions of group, time, and baseline depression and anxiety symptoms. Participant-level covariates of baseline age and baseline week gestation were also included to ensure effects were due to the app and not differences in age or weeks gestation. The random effect was participant intercept. We progressively removed nonsignificant covariates and higher-order terms and reran the model. We checked the model residuals to ensure conformance to model assumptions of normality and calculated the proportion of variance in the dependent variable attributable to the fixed effects.

## Results

Out of the 17 participants, 59% (n=10) had CAD and 41% (n=7) had MDD at baseline. Participants were primarily White, heterosexual, married, employed, and educated, with private health insurance, as seen in Table 1.

**Table 1.** Demographics and participant characteristics.

		Comorbid anxiety and depression (n=10)	Major depressive disorder (MDD; n=7)	Total (N=17)
Age (years), mean (SD)		33.4 (3.0)	35.8 (2.7)	34.4(3.1)
Weeks gestation, mean (SD)		18.1 (8.4)	15.1 (5.9)	16.8 (7.3)
**Race or ethnicity, n (%)**				
	White	10 (100)	2 (29)	12 (71)
	Black or African American	0 (0)	3 (43)	3 (18)
	Asian	0 (0)	2 (29)	2 (12)
	Non-Hispanic or Latina	9 (90)	6 (86)	15 (88)
	Hispanic or Latina	1 (10)	1 (14)	2 (12)
**Sexual orientation, n (%)**				
	Heterosexual	7 (70)	7 (100)	14 (82)
	Bisexual	2 (20)	0 (0)	2 (12)
	Queer	1 (10)	0 (0)	1 (6)
**Marital status, n (%)**				
	Married	9 (90)	3 (43)	12 (71)
	Divorced	0 (0)	1 (14)	1 (6)
	Never married	1 (10)	3 (43)	4 (24)
**Employment status, n (%)**				
	Employed	7 (70)	7 (100)	14 (82)
	Student	3 (30)	0 (0)	3 (18)
	Disabled or unable to work	0 (0)	0 (0)	0 (0)
**Insurance status, n (%)**				
	Private health insurance	10 (100)	6 (86)	16 (94)
	Medicaid	0 (0)	1 (14)	1 (6)
**Education level, n (%)**				
	Postgraduate training	7 (70)	5 (71)	12 (71)
	Bachelor’s degree	3 (30)	1 (14)	4 (24)
	Some college	0 (0)	0 (0)	0 (0)
	High school diploma	0 (0)	0 (0)	0 (0)
	Some high school	0 (0)	1 (14)	1 (6)
**Treatment, n (%)**				
	Psychiatric medication in the last 2 months	6 (60)	2 (29)	8 (47)
	Psychosocial treatment in the last 2 months	5 (50)	3 (43)	8 (47)

Table 1 shows the demographics and participant characteristics of our participants in a pilot study conducted between 2021 and 2022 in the United States testing whether baseline symptom and diagnostic differences influence the symptom trajectory of pregnant individuals using a BA gaming app for perinatal depression and anxiety.

There were no significant group differences in age (mean_CAD_ 33.4, SD_CAD_ 3.0 years and mean_MDD_ 35.8, SD_MDD_ 2.7 years), baseline weeks gestation (mean_CAD_ 18.1, SD_CAD_ 8.4 and mean_MDD_ 15.1, SD_MDD_ 5.9), or baseline depression symptoms (mean_CAD_ 12.8, SD_CAD_ 3.3 and mean_MDD_ 11.7, SD_MDD_ 3.5). There was no significant difference in baseline past 2-week anxiety symptoms (mean_CAD_ 10.7, SD_CAD_ 2.7 and mean_MDD_ 7.0, SD_MDD_ 2.7; Table 2). That is, while both diagnostic groups started with similar past 2-week levels of anxiety, only the CAD group met full *DSM-5* criteria for GAD, which requires a duration of at least 6 months of symptoms. The CAD group was 3.6 times more likely to be using psychotropic medications (6 out of 10, 60% of participants with CAD, 2 out of 7, 29% of participants with MDD) though with this sample size, it did not reach statistical significance (*P*=.32). The CAD group was 1.2 times more likely to be in therapy (5 out of 10, 50% participants with CAD and 3 out of 7, 43% participants with MDD); findings did not reach significance (*P*=.63).

**Table 2.** Mean changes from baseline to follow-up in anxiety and depression for completers.

		PHQ-9^a^ (mean, SD)	GAD-7^b^ (mean, SD)
**Comorbid anxiety and depression**			
	Baseline (n=10)	12.8 (3.7)	10.7 (2.7)
	Baseline completers (n=7)	12.7 (3.9)	10.0 (2.9)
	Follow-up (n=7)	7.0 (3.4)	6.0 (2.6)
	Completers mean change score	−5.7 (4.3)	−4.0 (4.2)
**Major depressive disorder**			
	Baseline (n=7)	11.7 (3.5)	7.0 (2.7)
	Baseline completers (n=3)	13.7 (4.5)	7.3 (0.6)
	Follow-up (n=3)	7.7 (4.9)	9.7 (6.5)
	Completers mean change score	−6.0 (1.7)	2.3 (6.5)

a PHQ-9: Patient Health Questionnaire-9.

b GAD-7: Generalized Anxiety Disorder-7.

Of the 10 participants with CAD, 7 (70%) completed the last study assessment and of the original 7 participants with MDD, 3 (43%) completed the last study assessment. Baseline diagnosis (CAD vs MDD) was not a significant predictor of whether participants completed the last assessment (β=−1.36, SE 1.00; *z* score=−1.35; *P*=.18). There was also no significant difference in the number of days using the app between the CAD group and the MDD group (mean_CAD_ 18.0, SD_CAD_ 16.20; mean_MDD_ 11.6, SD_MDD_ 19.79; *t*_7.04_=0.62; *P*=.56; 95% CI −18.09 to 30.89). There was no significant difference in the number of tasks completed between the CAD group and the MDD group (mean_CAD_ 13.22, SD_CAD_ 14.07; mean_MDD_ 6.0, SD_MDD_ 12.86; *t*_9.08_=0.97; *P*=.36; 95% CI −9.55 to 23.99).

There was no significant difference between the depression symptom mean change score for completers from baseline to follow-up for the CAD group compared to the MDD group (mean_CAD_ −5.7, SD_CAD_ 4.3; mean_MDD_ −6.0, SD_MDD_ 1.7; *t*_8_=0.11; *P*=.92; 95% CI −5.8 to −6.4). There was also no significant difference in the anxiety symptom mean change score for completers from baseline to follow-up for the CAD group compared to the MDD group (mean_CAD_ −4.0, SD_CAD_ 4.2; mean_MDD_ 2.3, SD_MDD_ 6.5; *t*_8_=−1.87; *P*=.09; 95% CI −14.1 to 1.5).

**Figure 1.**
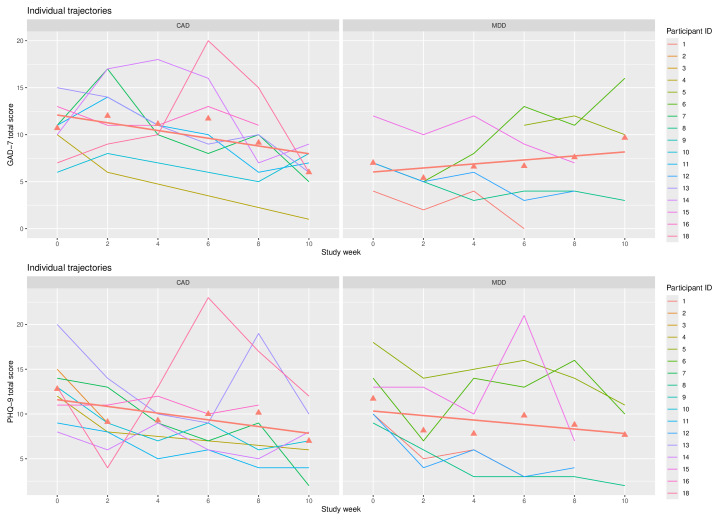
Individual symptom trajectories of anxiety (GAD-7) and depression (PHQ-9) by baseline diagnosis. Figure 1 shows raw score symptom trajectories for each participant on anxiety (GAD-7) and depression (PHQ-9) symptoms by baseline diagnosis in a pilot study conducted between 2021 and 2022 in the United States testing whether baseline symptom and diagnostic differences influence symptom trajectory in pregnant individuals using a behavioral activation gaming app for perinatal depression and anxiety. Red triangles denote the mean score at each study week, with the red line representing a least squares regression line fit to these means. CAD: comorbid anxiety and depression; GAD-7: Generalized Anxiety Disorder-7; MDD: major depressive disorder; PHQ-9: Patient Health Questionnaire-9.

When assessing individual-level trajectories of depression and anxiety symptom scores across study week by participant, we see within-group variation both for depression and anxiety symptom scores (Figure 1). In the MDD group, most individuals’ anxiety symptoms decreased over time, though 1 individual demonstrated an increase in anxiety. The anxiety of some individuals with CAD increased before decreasing over time. While overall depression symptoms decreased over time, a few individuals in both groups displayed increases in symptoms.

**Figure 2.**
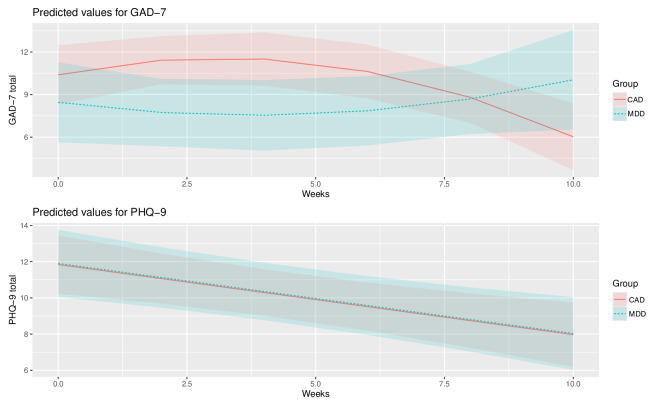
Model fixed effect predicted values of GAD-7 and PHQ-9 over time. Figure 2 shows effects plots from the 2 mixed effects models predicting anxiety (GAD-7; top panel) and depression (PHQ-9; bottom panel) across time in a pilot study conducted between 2021 and 2022 in the United States testing whether baseline symptom and diagnostic differences influence symptom trajectory in pregnant individuals using a behavioral activation gaming app for perinatal depression and anxiety. Predicted values from model fixed effects are shown with 95% confidence bands around the lines, controlling for baseline symptoms. CAD: comorbid anxiety and depression; GAD-7: Generalized Anxiety Disorder-7; MDD: major depressive disorder; PHQ-9: Patient Health Questionnaire-9.

For mixed effects models, nonsignificant higher-order terms and covariates were removed, and models rerun. Relevant to the assessment of any association of baseline diagnosis to change in anxiety across time controlling for baseline anxiety, there was a significant interaction between baseline diagnosis and quadratic study week (β=.18, SE 0.07; *t*_62_=2.61; *P*=.01). This interaction reflects a tendency for the CAD group anxiety symptoms to increase initially and then decrease at an accelerated rate, whereas the MDD groups’ anxiety symptoms were relatively stable across time or even showed a slight “U” shape pattern across time (Figure 2). Overall, the fixed effects accounted for 33.9% of the total variance in the anxiety symptom scores. All other fixed effects of interest were nonsignificant (Table 3).

For the assessment of baseline diagnosis of depression across time controlling for baseline depression, there was a significant effect of linear time on depression symptom scores (β=−.39, SE 0.11; *t*_68_=−3.51; *P*<.001), showing that depression symptoms decline steadily across time for both groups. We also found a significant effect of baseline diagnosis on depression symptoms at follow-up (β=−8.53, SE 3.93; *t*_13_=−2.17; *P*=.05), suggesting that being in the MDD group was associated with slightly higher follow-up depression symptom scores compared to the CAD groups’ follow-up depression symptom scores across time (Figure 2), holding other predictors (eg, baseline depression symptoms), constant. There was also a marginally significant interaction of baseline diagnosis with baseline depression symptoms (β=.65, SE 0.31; *t*_13_=2.12; *P*=.054) suggesting that the difference in follow-up depression scores by baseline diagnosis difference was slightly stronger among those with lower baseline depression levels than at higher levels.

**Table 3.** Mixed models fixed effects results. Table 3 shows fixed effects results from both mixed effects models in a pilot study conducted between 2021 and 2022 in the US testing whether baseline symptom and diagnostic differences influence symptom trajectory in pregnant individuals using a behavioral activation gaming app for perinatal depression and anxiety.

		β (SE)		*t* test (*df*)		*P* value
**GAD-7**^**a**^ **model**						
	Intercept	5.06 (2.62)		1.93 (15)		.07
	Baseline diagnosis	−1.94 (1.81)		−1.07 (32)		.29
	Time^b^	.75 (0.44)		1.72 (63)		.09
	Time^2^^c^	−.12 (0.04)		−2.76 (62)		.007
	Baseline GAD-7	.53 (0.23)		2.35 (13)		.04
	Baseline diagnosis time^b^	−1.24 (0.69)		−1.81 (62)		.08
	Baseline diagnosis time^2c^	.18 (0.07)		2.61 (62)		.01
**PHQ-9**^**d**^ **model**						
	Intercept	4.37 (2.52)		1.73 (12)		.11
	Baseline diagnosis	−8.53 (3.93)		−2.17 (13)		.049
	Baseline PHQ-9	.57 (0.18)		3.07 (11)		.01
	Time	−.39 (0.11)		−3.51 (68)		.001
	Baseline diagnosis baseline PHQ-9	.65 (0.31)		2.12 (13)		.054

a GAD-7: Generalized Anxiety Disorder-7.

b Time: linear time.

c Time2: quadratic time.

d PHQ-9: Patient Health Questionnaire-9.

## Discussion

### Principal Results

In this pilot study, our objective was to take a hypothesis-generating exploratory approach to assess differences in anxiety and depression symptom trajectories in participants with either CAD or MDD. While we expected to see less favorable depression and anxiety symptom trajectories (according to PHQ-9 and GAD-7, respectively) for CAD relative to the MDD group, our first main finding was that anxiety in the CAD group increased initially and then decreased at an accelerated rate, whereas in the MDD group, anxiety symptoms remained relatively stable across time despite no difference in baseline anxiety symptoms. Our other main finding was that depression symptoms declined steadily across time for both groups; however, those with MDD had higher follow-up depression scores compared to those with CAD, despite no differences in baseline depression symptoms. Given that there were no group differences in app engagement, these findings may not be explained by adherence or engagement. Our findings provide preliminary support for considering individual differences in baseline diagnostic characteristics when developing personalized mobile interventions, particularly in the context of treating perinatal individuals with psychological comorbidities.

With respect to the anxiety symptom trajectories, our findings are in line with the literature showing that compared to active controls, BA has a large effect on the reduction of depressive symptoms and a small effect on the reduction of anxiety symptoms when anxiety is secondary to depression [20]. In our study, it appears that the BA gaming app may be associated with a substantial reduction of anxiety symptoms for individuals with CAD. For these individuals, it may be that the BA components of the app target avoidance behaviors, which could result in a temporary increase in anxiety followed by reductions in anxiety potentially due to increased reward, habituation, and violation of expectations [6,21]. These findings suggest that the app may be especially effective for individuals with CAD, as it encourages in-the-moment ecologically relevant exposure to anxiety-provoking stimuli [7]. Particularly for individuals with CAD, for whom daily external stimuli may be especially provoking, the app may be especially useful and serve as an exposure.

Despite no significant group difference in baseline anxiety, the MDD group did not have a significant reduction in their anxiety symptoms across the study period, and some individuals had an increase in anxiety. Future studies should explore whether the anxiety experienced by the MDD group is maintained by other cognitive mechanisms to improve targeting anxiety symptoms when anxiety symptoms are secondary to depression symptoms. Findings may point to opportunities for the augmentation of BA gaming apps for those with MDD to more effectively target anxiety symptoms. Future treatment personalization studies should also assess whether notifying patients of common treatment trajectories based on baseline presentations may improve treatment adherence. Findings of individual variability in anxiety and depression over time point to an opportunity for future more highly powered studies investigating the risk for increasing anxiety and depression across treatment during pregnancy.

Regarding our hypothesis that the CAD group may have less favorable depression symptom trajectories, we instead found that depression symptoms decreased linearly across time for both groups and that the MDD group had higher depression symptom scores at follow-up compared to the CAD groups’ follow-up symptoms, suggesting that the app may be beneficial in reducing depression in perinatal individuals with different comorbidity profiles. These findings are in line with results from traditional and mobile BA interventions that BA is efficacious for the reduction of depressive symptoms (eg, [6,20,22]) even for individuals with different comorbidity profiles.

### Limitations

This pilot study has several limitations (eg, modest sample size and no control group; discussed further in Vanderkruik et al [14]). Future fully powered studies should consider app engagement when assessing differences in symptom trajectories by baseline diagnosis. Due to the small sample size and lack of a control group, the results should be interpreted with caution and be considered as hypothesis generating.

### Conclusions

The findings provide preliminary support for considering individual differences and comorbidities when developing scalable mobile interventions for perinatal populations. This pilot study has potentially important broad implications. Demand for treatment, particularly in perinatal populations, is high and yet can be hard to access for a variety of reasons, including the fact that many traditional interventions require presence in a formal and controlled therapy environment. This study represents an initial step toward developing scalable interventions and highlights the need to consider baseline differences in future studies that may personalize the app experience to increase adherence and effectiveness. Further research involving an adequately powered randomized controlled trial may further illuminate individual differences affecting the impact of mobile interventions.

## Abbreviations

BA: behavioral activation
CAD: comorbid anxiety and depression
*DSM-5*: *Diagnostic and Statistical Manual of Mental Disorders, Fifth Edition*
GAD: generalized anxiety disorder
GAD-7: Generalized Anxiety Disorder-7
MDD: major depressive disorder
MINI: Mini International Neuropsychiatric Interview
PHQ-9: Patient Health Questionnaire-9
